# Emerging epigenetic dynamics in gut-microglia brain axis: experimental and clinical implications for accelerated brain aging in schizophrenia

**DOI:** 10.3389/fncel.2023.1139357

**Published:** 2023-05-15

**Authors:** Benneth Ben-Azu, Elisabetta C. del Re, Jared VanderZwaag, Micaël Carrier, Matcheri Keshavan, Mohammadparsa Khakpour, Marie-Ève Tremblay

**Affiliations:** ^1^Division of Medical Sciences, University of Victoria, Victoria, BC, Canada; ^2^Department of Pharmacology, Faculty of Basic Medical Sciences, College of Health Sciences, Delta State University, Abraka, Nigeria; ^3^Department of Psychiatry, Harvard Medical School, Boston, MA, United States; ^4^VA Boston Healthcare System, Brockton, MA, United States; ^5^Beth Israel Deaconess Medical Center, Boston, MA, United States; ^6^Axe Neurosciences, Centre de Recherche du CHU de Québec, Université Laval, Québec City, QC, Canada; ^7^Department of Biochemistry and Molecular Biology, The University of British Columbia, Vancouver, BC, Canada; ^8^Department of Neurology and Neurosurgery, McGill University, Montréal, QC, Canada; ^9^Department of Molecular Medicine, Université Laval, Québec City, QC, Canada; ^10^Centre for Advanced Materials and Related Technology (CAMTEC), Institute on Aging and Lifelong Health (IALH), University of Victoria, Victoria, BC, Canada

**Keywords:** microglia, microbiome, epigenetics, schizophrenia, aging, psychobiotics, dysbiosis, vagus nerve

## Abstract

Brain aging, which involves a progressive loss of neuronal functions, has been reported to be premature in probands affected by schizophrenia (SCZ). Evidence shows that SCZ and accelerated aging are linked to changes in epigenetic clocks. Recent cross-sectional magnetic resonance imaging analyses have uncovered reduced brain reserves and connectivity in patients with SCZ compared to typically aging individuals. These data may indicate early abnormalities of neuronal function following cyto-architectural alterations in SCZ. The current mechanistic knowledge on brain aging, epigenetic changes, and their neuropsychiatric disease association remains incomplete. With this review, we explore and summarize evidence that the dynamics of gut-resident bacteria can modulate molecular brain function and contribute to age-related neurodegenerative disorders. It is known that environmental factors such as mode of birth, dietary habits, stress, pollution, and infections can modulate the microbiota system to regulate intrinsic neuronal activity and brain reserves through the vagus nerve and enteric nervous system. Microbiota-derived molecules can trigger continuous activation of the microglial sensome, groups of receptors and proteins that permit microglia to remodel the brain neurochemistry based on complex environmental activities. This remodeling causes aberrant brain plasticity as early as fetal developmental stages, and after the onset of first-episode psychosis. In the central nervous system, microglia, the resident immune surveillance cells, are involved in neurogenesis, phagocytosis of synapses and neurological dysfunction. Here, we review recent emerging experimental and clinical evidence regarding the gut-brain microglia axis involvement in SCZ pathology and etiology, the hypothesis of brain reserve and accelerated aging induced by dietary habits, stress, pollution, infections, and other factors. We also include in our review the possibilities and consequences of gut dysbiosis activities on microglial function and dysfunction, together with the effects of antipsychotics on the gut microbiome: therapeutic and adverse effects, role of fecal microbiota transplant and psychobiotics on microglial sensomes, brain reserves and SCZ-derived accelerated aging. We end the review with suggestions that may be applicable to the clinical setting. For example, we propose that psychobiotics might contribute to antipsychotic-induced therapeutic benefits or adverse effects, as well as reduce the aging process through the gut-brain microglia axis. Overall, we hope that this review will help increase the understanding of SCZ pathogenesis as related to chronobiology and the gut microbiome, as well as reveal new concepts that will serve as novel treatment targets for SCZ.

## 1. Introduction

Schizophrenia (SCZ) is a serious disorder affecting 1% of the population worldwide that poses devastating consequences for the individuals affected but also society (Velligan and Rao, 2023). The estimated cost to society (2013) is approximately $155 billions (Cloutier et al., 2016). A recent Danish study has shown that healthcare costs for chronic SCZ is estimated to be up to 10 times higher than the cost for other chronic neurological diseases such as epilepsy or multiple sclerosis (Hastrup et al., 2020); this difference in cost is explained in part by the high number of co-morbidities accompanying SCZ (Wilson et al., 1998; Hennekens et al., 2005). However, SCZ is characterized by complex and diverse symptoms which are widely grouped into positive (e.g., hallucination, delusion, thought disorder), negative (e.g., asociality, anhedonia, amotivation), and cognitive (e.g., loss of learning and memory functions) (Keshavan et al., 2011b; Ben-Azu et al., 2016, 2018a,b; Blokland et al., 2017; Okubo Eneni et al., 2020; Ishola et al., 2021).

The genetic contribution to developing SCZ is relatively high, with heritability estimates of 81% by meta-analysis of twin studies and 64% by a large family based study (Sullivan et al., 2003). Genome wide association studies have greatly contributed to an understanding of the highly polygenic genetic structure of SCZ (Sekar et al., 2016; Hudson and Miller, 2018; Dennison et al., 2020). Nevertheless, each common genetic variants only has a small effect. Genetic studies robustly indicate that many of the single nucleotide polymorphisms (SNPs) conferring an increased risk for SCZ are shared with other neurodevelopmental disorders (Karimian et al., 2020; Guo et al., 2021) and linked to genes that are important for neural migration and proliferation (Walsh et al., 2008; Karimian et al., 2020; Song et al., 2022). Abnormalities among several brain regions were also identified (Ohtani et al., 2018; Del Re et al., 2021), yet the neural mechanisms underlying the disorder are largely unknown. In addition, SCZ and other mental disorders are often accompanied by serious prodromal co-morbidities (Newcomer, 2007; Kirkpatrick et al., 2008; Kirkpatrick and Kennedy, 2018; Penninx and Lange, 2018; Mazereel et al., 2020) including cardiovascular diseases (Wilson et al., 1998; Goff et al., 2005; Hennekens et al., 2005; Demaria et al., 2014), metabolic disorders and inflammatory bowel diseases (IBDs) (Newcomer, 2007; Kashani et al., 2017; Bernstein et al., 2019; Verdugo-Meza et al., 2020).

There are multiple conceptualizations of SCZ that are not necessarily exclusive. As more data illustrate the biological timeline and mechanisms of the disease, SCZ is increasingly painted as both a neurodevelopmental disease and a disease of accelerated aging. The conceptualization of SCZ as a neurodevelopmental disorder demonstrates the complexity of the disease as a whole (Murray and Lewis, 1987; Keshavan and Paus, 2015; Murray et al., 2017; Ben-Azu et al., 2022). Whereas SCZ symptomatology most often emerges in late adolescence, phenotypic alterations can arise much earlier in childhood before the onset of symptomatology into adulthood (Woodberry et al., 2008; Sørensen et al., 2010). Accompanying these neurodevelopmental SCZ concept, there is a growing body of experimental data identifying SCZ as a disease of accelerated aging (Okusaga, 2013; Kirkpatrick and Kennedy, 2018; Nguyen et al., 2018; Teeuw et al., 2021). Being affected by SCZ predicts a shorter lifespan, by 10–15 years compared to the general population (Kirkpatrick et al., 2008), with a mortality rate that is ∼10 times higher than in age-matched controls (Lindqvist et al., 2015). An autopsy-based study showed that 77.8% of admitted decadents died of sudden cardiac death such as myocarditis, cardiomyopathy, coronary artery atherosclerosis, and pulmonary thromboembolism (Chen et al., 2022). However, other causes of death were also mentioned, including respiratory inflammation and hepatic steatosis (Chen et al., 2022), as well as antipsychotic type such as risperidone and flupentixol owing to their blocking effects of cardiac potassium channel encrypted by the human Ether-à-go-go-related gene (hERG) (van Noord et al., 2011; Chen et al., 2020). One pathomechanistic reason identified between the increased death rates, and cardiovascular dysfunctions, was suggested to include the presence of altered levels of the immune-active gut-bacterial derived metabolite, trimethylamine *N*-oxide (TMAO), notably involved in the exacerbation of neuropsychiatric diseases characterized by vascular senescence, reduced capacity to regenerate hematopoietic system, and accelerated aging (Zeisel and Warrier, 2017; Ke et al., 2018; Li et al., 2018; Chen et al., 2019; Brunt et al., 2020). Of note, other measures linked to accelerated aging were examined, including shortening of telomere length (Zhang et al., 2016; Whittemore et al., 2019). Also, increased inflammation and oxidative stress (Olivieri et al., 2018), which are markers of cellular senescence, are consistent with the shortened telomere length. Some of these findings also strongly corroborate a tight link between inflammatory processes and aging characterized by a decreased capacity to regenerate the hematopoietic system (Lindqvist et al., 2015; Leboyer et al., 2016; Zhang et al., 2016). Additionally, an increased rate of telomere shortening processes was shown in major psychiatric disorders such as SCZ (Lindqvist et al., 2015), where microglia-derived pro-inflammatory markers are increased in the central nervous system (CNS) (Young et al., 2014; Leboyer et al., 2016; Lizano et al., 2021). Robust evidence also reveals increased rate of telomere shortening in leukocytes across major psychiatric illnesses that include SCZ, thus corroborating a tight link between inflammatory processes and aging (Lindqvist et al., 2015; Leboyer et al., 2016; Zhang et al., 2016); with a positive relationship between the length of illness and levels of CNS inflammation, telomere shortening, and oxidative stress (Nguyen et al., 2018).

The immune system is intimately connected to the gut microbiota, a system composed of ∼40,000 bacterial species (Sender et al., 2016) that was additionally shown to tightly affect behavior [see review by (Cryan and Dinan, 2012)]. The gut microbiota was experimentally demonstrated to mirror the aging process as its composition reflects shifting biological age (Galkin et al., 2020; Mullin et al., 2020). In individuals affected by SCZ, there is evidence of a unique gut microbiome composition compared to age-matched controls (Shen et al., 2018), suggesting a tight connection between cellular and chronological aging, gut microbiome, immunity, and microglial reactivity. Microglia, which are yolk-sac derived tissue-resident macrophages, are the brain’s immune sentinels, responsive to aging, trauma, injury, infection and diseases (Erny et al., 2015; Bisht et al., 2016; Hong and Stevens, 2016). Microglia actively maintain brain homeostasis in both steady-state and pathology via a variety of cellular and molecular mechanisms (Tremblay et al., 2010; Tay et al., 2017). Microglia regulate synapses by eliminating axonal fragments, terminals and dendritic spines (Tremblay et al., 2010; Tay et al., 2017). Gut microbiome-derived antigenic materials that influx the CNS are also known to be eliminated by microglia (Tay et al., 2017). In the following sections, we progressively summarize evidence for the conceptualization of “gut-brain microglia axis” hypothesis. Given the heterogeneous pathogenesis of SCZ, our review was aimed to bring together authors from diverse backgrounds in order to provide a broad discussion of the various biological substrates of the disease. Therefore, we discuss how dysbiosis affects microglial function in SCZ and the accelerated brain aging linked to the disease. We also debate the outcome of antipsychotic drugs on the gut microbiome followed by evidence showing that psychobiotics might contribute to antipsychotic-induced therapeutic benefits or adverse effects via a modulation of the gut-brain microglial axis.

## 2. Brain reserve and morphogenesis in SCZ

Reports from neuroimaging studies have provided mounting evidence for structural and functional abnormalities of brain reserves in SCZ (Keshavan et al., 2011a). Here the concept of brain or neuronal reserve especially of cortical origin can be considered as the brain architecture that prevents the development or expression of a neuropsychiatric condition or delay the occurrence of premature aging (Shenton et al., 2001; Stern et al., 2019; Del Re et al., 2021). According to Stern’s extensive conception (Stern et al., 2019), brain reserve can be conceptualized as the brain resources that allow some individuals to better withstand pathological processes and healthy aging. The brain reserve includes morphometry, such as cortical thickness (CT), surface area (SA), volume, number of neurons and/or other neuro-biological factors. Cognitive reserve (CR) is an additional measure which according to Stern et al. (2019) reflects the cognitive flexibility of the brain exposed to day-to-day life events, as well as pathologies and aging. In studies of patients with SCZ, higher brain reserve, measured as greater surface area and gray matter, was predictive of both social and cognitive responses to Cognitive Enhancement Therapy (Keshavan et al., 2011b), indicating a role of the brain reserve in the pathophysiological course of SCZ. While high cortical reserves particularly in the temporal cortex and superior temporal gyrus gray matter were linked to improved social cognitive response, a low cortical reserve was hypothesized as a risk mediating factor for many forms of mental illness (Shenton et al., 2001; Keshavan et al., 2011a).

In SCZ, the CT and SA components of the cortex develop along distinct developmental pathways which are mostly genetically unrelated and follow differentiated morphogenetic stages during cortical formation (Lichtenstein et al., 2009; Rimol et al., 2012). The bulk of the cortical structure development is completed prenatally while increased gyrification of the superior and inferior frontal gyri is indicative of further postnatal development (Rimol et al., 2012; Del Re et al., 2021). During neurodevelopment, SA and CT interact dynamically and increase during the first years of life (Gilmore et al., 2012; Lyall et al., 2015). Longitudinal data indicate a non-linear maturation of gray matter density, a measure that includes both SA and CT (ages 4–21 years) (Gogtay et al., 2004), while higher order association cortices mature significantly later than lower-order ones (Gogtay et al., 2004). Within the temporal lobe, the superior and inferior temporal gyri exhibit slowest maturation, continuing up to age ∼20–21. Within the superior temporal gyrus, the posterior area appears to mature last (Gogtay et al., 2004). Other longitudinal studies of CT (Sowell et al., 2004) (ages 5–11 years) or CT and SA (age 7–29 years) (Tamnes et al., 2014; Fjell et al., 2015; Ducharme et al., 2016) indicate extended fine-tuning of neuronal connections far beyond childhood, especially in language-related cortices. The prefrontal cortex, essential to executive function, might be the last region to mature (Paus et al., 2008). Cognition and other complex functions are associated with an intact cortex and ultimately genetics influences the expansion of SA and CT along specific directions (Grove and Fukuchi-Shimogori, 2003; Narr et al., 2005; Stiles and Jernigan, 2010; Alexander-Bloch et al., 2013). In SCZ, smaller CT is described in the prefrontal, temporal, parietal and occipital regions at various stages of disease progression (Narr et al., 2005; van Haren et al., 2011; Rimol et al., 2012; Cannon et al., 2015). An association of specific symptomatology, positive vs. negative symptoms, with temporal (Walton et al., 2017) and prefrontal (Walton et al., 2018) CT, respectively, was also shown. In SCZ (Blokland et al., 2017) and other neurodevelopmental disorders (Schubert et al., 2015), there is a tight genetic relationship between cognitive dysfunction and disease vulnerability (del Re et al., 2014; Toulopoulou et al., 2015; Blokland et al., 2017; Song et al., 2022). There is further an association, albeit less clear, between SCZ and cortical characteristics, especially CT (Cannon et al., 2015).

While the process of morphogenesis is especially important in the study of neurodevelopmental disorders, as it determines the overall structure of the cortex and the relationships between its regions, SCZ was also described as a disease of accelerated aging (Nguyen et al., 2018). Conceptualizing the difference between chronological aging and biological aging (He and Sharpless, 2017) is important in interpreting the epidemiology of SCZ and other serious mental diseases (Nguyen et al., 2018). Cellular senescence (He and Sharpless, 2017), as part of the aging process, includes cellular growth arrest and activation of several cellular pathways that respond to DNA damage (Campisi, 2005). Senescent cells accumulate with increasing age; this process is possibly linked to lowered immune clearance (Demaria et al., 2014; Muñoz-Espín and Serrano, 2014; Sanoff et al., 2014), increased production of senescent cells themselves, abnormal DNA repair, and telomere dysfunction (Lindqvist et al., 2015; Zhang et al., 2016). Telomeres, sequences of repetitive DNA at the end of chromosomes, are shortened under conditions of sustained DNA damage (Whittemore et al., 2019). Consequently, senescence markers accumulate in several tissues including the CNS in humans and animal models during healthy aging itself (Molofsky et al., 2006; Sousa-Victor et al., 2014). Molecular senescence can be linked to immune cell phenotypes such as dystrophic microglia that are also characteristic of the aging process (He and Sharpless, 2017; Candlish and Hefendehl, 2021; Shahidehpour et al., 2021).

## 3. Evidence of accelerated aging in SCZ

Growing evidence support an accelerated aging and cognitive decline process in SCZ (Kirkpatrick et al., 2008; Okusaga, 2013; Schnack et al., 2016; Islam et al., 2017; Stone et al., 2020). This probable endophenotype of SCZ is described by different hallmarks (Carrier et al., 2021). There is evidence that SCZ and accelerated aging are linked to changes in epigenetic clocks (Teeuw et al., 2021) and that accelerated brain aging in SCZ significantly occurs around the period of first episode psychosis leading to an average 5.5 years older brain biological vs. chronological age (“brain age gap”) (Koutsouleris et al., 2014; Schnack et al., 2016; Hajek et al., 2019; Kaufmann et al., 2019; Shahab et al., 2019). The trajectory of brain aging can be predicted based on evidence from neuroimaging of decreased gray matter volume (Cole and Franke, 2017; Cole et al., 2017) and inverted U-shape curve white matter (Mwangi et al., 2013), as well as the inter-organ activities (Lai et al., 2021; Nguyen et al., 2021). Of note, age-dependent depreciation of both the gray and white matter function has been recorded to occur in males vs. females, which supports the view that SCZ is a sexually dimorphic disease with males showing increased derangement in brain reserve and cognitive decline compared to female counterparts (Lee et al., 2020). Thus, several clinical reports revealed decreased cognitive features such as reduced information processing speed, vigilance/attention, and social flexibility which were age-dependent and differed between males and females, suggesting gradual degenerative processes (Lee et al., 2020). One possible explanation that could be provided for this cognitive decline of SCZ patients is accelerated brain aging (Sheffield et al., 2016; Shahab et al., 2019). Accordingly, investigation with diffusion tensor imaging was performed to show a profound reduction in the leftward asymmetry among some key white matter areas in SCZ (Ribolsi et al., 2014). This abnormal functional connection and asymmetry of intra-hemispheric connectivity in the brain of patients with SCZ is attributable to the structural impairment and loss of inhibition across the corpus callosum (Ribolsi et al., 2014). Notably, this attenuated left-right asymmetry has been reported to play key roles in determining disease progression and major psychotic symptoms such as loss of reality-based belief, altered perception integration and attentional surveillance as well as core cognitive deficits of SCZ (Rentería, 2012; Ribolsi et al., 2014; Zhang et al., 2015; Gurin and Blum, 2017). Recent cross-sectional brain magnetic resonance imaging (MRI) in patients with SCZ (*N* = 715 scans, mean scan interval of 3.4 years) and blood sample analyses based on two epigenetic age clocks (*N* = 172) examining DNA methylation age (DNAmAge; measure of cellular aging, but not senescence) and phenotypic age (phenoAge; measure that captures all risk factors of morbidity and mortality) gaps revealed a connection between SCZ and accelerated biological aging (Teeuw et al., 2021). The study reported that patients with SCZ presented signs of accelerated age-related decline in cognition based on decreased gray matter and physiological domains. They also found reduced brain reserves with an increased mortality due to cardiovascular issues based on altered metabolism compared to normally aging individuals. The authors found that polygenic risk of patients with SCZ matches an accelerated brain aging yet correlates negatively with the DNAmAge contrary to the phenoAge metrics. This finding supports the view that the accelerated aging rate observed in these patients implies a distinct biological process (Levine et al., 2018; Teeuw et al., 2021).

Further, cellular aging can be assessed using telomere length, where SCZ patients have shorter telomeres compared to healthy subjects (Czepielewski et al., 2016; Omidpanah et al., 2019). Follow-up investigation from one of the groups showed similar results while also correlating with the decreased brain gray matter volume measured using MRI described by other groups (Czepielewski et al., 2018; Carrier et al., 2020). It is important to mention though that the findings appear conflictual across studies [see for example (Omidpanah et al., 2019)], possibly due to differences in medication or subsets of patients with SCZ. Another hallmark of cellular aging measured in SCZ patients is oxidative stress (Okusaga, 2013). When cognitive functions were measured using the Repeated Battery for the Assessment of Neuropsychological Status and correlated with super oxide dismutase activity in the blood of patients, a significant negative correlation was found (Wang et al., 2021), supporting the involvement of oxidative stress in mediating cognitive decline (Ben-Azu et al., 2018c,d, 2022). Using DNA methylation as a proxy for cellular aging, two studies also showed no acceleration of cellular aging in SCZ patients (McKinney et al., 2017; Voisey et al., 2017). However, a more recent study found evidence of DNA methylation in patients with SCZ, in a cohort-dependent manner (Okazaki et al., 2019). Using two different sets of patients consisting of hospitalized chronic long-term vs. medication-free SCZ patients, the study revealed a decreased extrinsic epigenetic age acceleration (EEAA) in the blood of patients with long-term SCZ contrary to the medication free group. The study provided evidence showing the correlation between DNAm age and chronological aging. However, no changes were observed in the intrinsic epigenetic age acceleration for both groups (Okazaki et al., 2019), indicating the implication of EEAA in driving DNA methylation and accelerated aging. Taking multiple aging hallmarks together [i.e., telomere length, blood levels of oxidative stress, C-C motif chemokine (CCL)-11 and 24], a machine learning algorithm was able to distinguish SCZ patients from controls in 80% of cases vs. in only 62.5% of cases when comparing SCZ patient with their siblings (Rebouças et al., 2021). This highlights the role of accelerated aging in SCZ and other related neurodevelopmental diseases, while the SCZ aging risk may be shared among siblings. Overall, including brain volume measurements determined with MRI in these analyses could allow to portray significantly more accurate distinctions from controls.

## 4. Microglia and their sensomes

Microglia are the immune cells dedicated to the protection of the CNS (Ginhoux et al., 2010; Colonna and Butovsky, 2017; Kabba et al., 2018). Microglia play vital roles in development, homeostasis and remodeling, including via neurogenesis, synaptic formation and elimination, as well as myelination [reviewed in (Salter and Beggs, 2014; Hong and Stevens, 2016; Tay et al., 2017; Hughes and Appel, 2020; Tremblay, 2021)]. Notably, microglia are involved in brain rewiring via the phagocytosis of less active synapses (Sierra et al., 2010; Tremblay et al., 2010; Schafer et al., 2012). Furthermore, microglia can eliminate synapses in a process named synaptic stripping, where their dynamic processes physically separate synaptic elements (Trapp et al., 2007). These roles are crucial for proper brain development and plasticity, and require a constant neuron-microglia communication (Tremblay et al., 2011; Eyo and Wu, 2013; Szepesi et al., 2018). Microglia achieve this feat using their sensomes, which are groups of proteins and binding sites that facilitate their interactions with neurons, among other cell types (Hickman et al., 2013). These interactions enable microglia to fulfill their physiological and immune functions in health and diseases (Carrier et al., 2021). Microglia-neuron communication is notably mediated via the fractalkine receptor CX3CR1 (Harrison et al., 1998; Ransohoff and Perry, 2009; Paolicelli et al., 2014; Lauro et al., 2019; Tremblay, 2021). This receptor, but also the complement receptors, triggering receptor expressed on myeloid cells 2 and purinergic receptors are all involved in neuron-microglia signaling and are central for synaptic pruning and phagocytosis (Schafer et al., 2012; Zhan et al., 2014; Arnoux and Audinat, 2015; Sipe et al., 2016; Filipello et al., 2018; Gunner et al., 2019). Microglia further express tyrosine kinase receptors like Tyros3, Axl, and Mer (TAM) which are important for neuronal cell removal in health and diseases such as Parkinson’s disease (Fourgeaud et al., 2016). Microglia act as a damage sensor for the CNS via TAM-regulated activity, as microglia in TAM-deficient mice exhibit decreased motility and attraction to sites of injury (Fourgeaud et al., 2016). However, microglial function is altered upon excessive release of pro-inflammatory cytokines, as well as activation of the complement pathway in different brain areas (Fekete et al., 2019). In SCZ, one of the strongest genetic associations is with the locus at the major histocompatibility complex, originating from alleles of the complement component C4 (Sekar et al., 2016). In animal models, C4 has a central role in microglia-mediated synapse elimination (Yilmaz et al., 2021), providing a direct evidence for an immune system involvement in SCZ pathophysiology (Sekar et al., 2016). C4 polymorphism in microglia was linked to an up-regulation of pro-inflammatory markers including c-reactive protein, interleukin (IL)1β and IL-8 (Hepgul et al., 2012; Chiappelli et al., 2017; David et al., 2017), as well as strong levels of Nod-like receptor protein 3 (NLRP3) inflammasome in the brain and blood of a subset of SCZ patients (Scheiblich et al., 2017; Ventura et al., 2020). There are evidence that inflammation and accelerated aging have a complex link, particularly in the context of stress and mental health (Ben-Azu et al., 2020; Carrier et al., 2021). Chronic inflammation can induce telomere shortening leading to increased aging process (Jurk et al., 2014) or “inflammaging.” Inflammaging is characterized by a chronic inflammation accelerating the brain aging process where the brain immune system is highly involved, including microglia (Franceschi and Campisi, 2014; Franceschi et al., 2018, 2007).

## 5. Gut-brain axis

**FIGURE 1:**
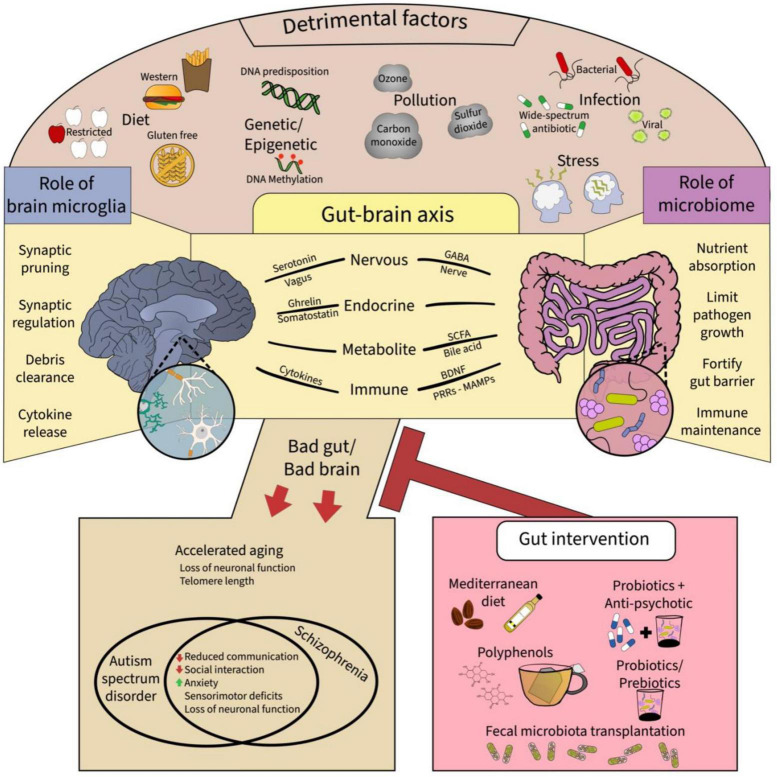
A summary of the epigenetic factors and putative extracellular mechanisms that provide communicative pathways for gut-brain microglia axis. Top panel shows the myriad of detrimental factors affecting the brain and the gut, which include the diet, genetic and epigenetic changes, pollution, infection, and stress. These factors affect the gut-brain axis, shown in the middle panel, via respective roles particularly affecting the gut microbiome and brain microglia. Ultimately, adverse effect on the gut-brain microglia axis can result in neurodevelopmental disorders and accelerated aging in the offspring which might be mitigated by gut interventions such as the Mediterranean diet, polyphenols, probiotics with antipsychotics, and fecal microbiota transplantation, all promoting microbiome diversity and proper function. GABA: gamma amino butyric acid, BDNF: brain-derived neurotrophic factor, MAMPS: microbe-associated molecular patterns, PRRs: pathogen recognition receptors.

The gut-brain axis (GBA) is a term used to describe the bidirectional relationships between the gastrointestinal (GI) tract and CNS (Figure 1). The GI tract is home to a plethora of microorganisms including bacteria, fungi, viruses, archaea, and protozoa (Morais et al., 2021). Gradually from infancy to adulthood, the composition of the gut microbiome is established, from initial maternal microbiota exposure (Yao et al., 2021) to subsequent environmental inputs (Dominguez-Bello et al., 2019; Qi et al., 2021). Generally, the vast ecosystem inhabiting the GI tract performs three main functions to the benefit of both microorganisms and host (Ducarmon et al., 2019; Qi et al., 2021). The first main function is nutrient absorption, particularly of substances typically not digestible by the human GI tract alone. Second, via nutrient absorption, gut microorganisms create a competitive environment that drastically limits pathogen colonization. Third, gut microorganisms fortify the gut via secretion of trophic factors strengthening epithelial barriers. Additionally, recent research has revealed a fourth critical function of the gut microbiome in the development, maturation and maintenance of the immune system including microglia (Morais et al., 2021). Importantly, the gut microbiota communicate with the brain via vagal innervation (Forsythe et al., 2014; Kaczmarczyk et al., 2017). The vagal nerve projects to the brainstem locus coeruleus through which the cholinergic and noradrenergic systems connect different brain regions, including the nucleus basalis of Meynert, via cholinergic and noradrenergic receptors which are notably expressed on microglia (Kaczmarczyk et al., 2017; Wang et al., 2018). Research into this topic demonstrates GBA’s critical role in growth and development of the host, indicating that disruptions to the microbiota balance can have devastating and global effects for the host including brain health (Erny et al., 2015, 2021). Overall, recent research has demonstrated the critical role that the GBA plays in the development and progression of neuropsychiatric disorders such as, autism spectrum disorder (ASD), mood disorders, and SCZ, making the microbiome a very promising novel area of therapeutic intervention. In the next section, the contributions that the GBA make toward neurodevelopment are on focus.

## 6. Evidence of gut-brain microglia axis connection

The connection of the gut to the brain is a relatively recent finding, with contemporary research revealing high associations between negative gut health and psychological conditions. While these associations are quite profound, this field is still elucidating the biological mechanisms underlying these complex relationships. Excitingly, many studies are demonstrating promising therapeutic interventions. In this section, a brief overview of research associating the gut microbiota and their microbiome with psychological disorders will be provided, followed by a discussion of mechanisms underlying this gut-brain communication.

A distinct association appears to be present between GI tract disorders, affective disorders, and cognitive dysfunction. Multiple studies have demonstrated high correlations between GI disorders, anxiety trait and state, depressive symptoms, and even personality differences (Tosic-Golubovic et al., 2010; Bercik et al., 2011; Heijtz et al., 2011; Schmidtner et al., 2019). Another study revealed that children with (vs. without) GI disorders are more likely to present increased symptom severity on measures of irritability, social withdrawal, and anxiety (Nikolov et al., 2009). Similar findings were obtained in children with ASD (Mazefsky et al., 2014), while gut microbiota transferred from humans with ASD to mice triggered the onset of ASD-related behaviors in an animal model (Sharon et al., 2019). Further, evidence emphasize that the microbiota can underlie cognitive dysfunction and affective disorders in patients with GI tract disorders (Addolorato et al., 2008; Morais et al., 2021). Research is overall increasingly supporting the importance of modulating the GBA to treat many neuropsychiatric disorders including SCZ (Wang and Kasper, 2014; Ding et al., 2021). Although the biological means underpinning the microbiome’s neurological effects are not fully understood in humans, preclinical research is gradually shedding light onto these mechanisms.

Various mechanisms connecting the gut and brain involve a combination of nervous, endocrine, metabolic, and immune communication pathways (Clapp et al., 2017). The GI tract and its microbiome are responsible for the digestion of food, from which the body and brain are provided with energy and other chemical building blocks like amino acids and vitamins required for optimal function (Clapp et al., 2017). Although it is difficult to fully isolate metabolic effects of the gut microbiota from the effects of other systems, such as nervous or endocrine, the evidence points toward the metabolic properties of the gut microbiome as critical for CNS health and disease (Kamdar et al., 2016; Yu et al., 2017). Many neurotransmitters and their precursors are produced in the gut by certain strains of microorganisms. For example, *Bacteroides*, *Bifidobacterium*, *Escherichia*, and *Lactobacillus* spp. produce gamma-aminobutyric acid (GABA), the major inhibitory neurotransmitter implicated in SCZ pathogenesis (Barrett et al., 2012; Strandwitz et al., 2019; Patrono et al., 2021). Oral administration of these microbial species in mouse models demonstrated increased serum levels of GABA and brain levels of GABA_*A*_ receptors (Bravo et al., 2011; Strandwitz et al., 2019; Qi et al., 2021). Other microbial metabolites generated by the gut microbiota, such as bile acid and methylamine N-oxide, are critical for host development (Qi et al., 2021). Further, the enteroendocrine cells (EEC) are specialized secretory cells found across the stomach, pancreas, and GI tract which secrete various hormones in response to stimulation (Qi et al., 2021). Among these hormones are ghrelin and somatostatin, which are both critical to appetite regulation and exert a global effect on metabolism and growth (Qi et al., 2021). Additionally, EECs are responsible for serotonin secretion in the gut (Yu and Li, 2022). However, serotonergic dysregulation, particularly in the hippocampus, a highly plastic region linked to cognitive dysfunction and other behavioral deficits, has been consistently reported in the pathogenesis of SCZ (Chatterjee et al., 2012; Ben-Azu et al., 2018b,^2023^). This disruption could, in part, be linked to elevated levels of serotonin and its primary metabolite as observed in the hippocampus of germ free (GF) male mice (Clarke et al., 2013; Yano et al., 2015; Morais et al., 2021). Also, increased serum concentration of tryptophan, the precursor agent for serotonin synthesis was reported in GF mice (Clarke et al., 2013). These findings thus suggest possible humoral mechanisms through which the microbiome could regulate central pathways dependent on the serotonergic system. Furthermore, it was observed in mice that changes in gut microbiome alter levels of brain-derived neurotrophic factor (BDNF), a protein that is highly associated with synaptic plasticity and neurogenesis (Bercik et al., 2011; Bistoletti et al., 2019). Several studies further demonstrated reduced synaptic proteins alongside BDNF and impaired neurogenesis in patients with SCZ and IBD (Bercik et al., 2011; Szeligowski et al., 2020). These chemical messengers, crucial for healthy brain function, are an output of a healthy gut, demonstrating the reliance of the brain on outside systems to ensure its performance. Thus, these findings summarized that the intrinsic systems involved in neurochemical transmission and neuronal development are indeed affected by changes in gut microbiota diversity.

Another system which is important to the biological mechanisms underlying the connection of the gut to the brain is the enteric nervous system (ENS), the GI tract’s nervous system. The ENS, regulates gut activities such as peristalsis, permeability, and nutrient absorption (Furness, 2012; Heiss and Olofsson, 2019; Joly et al., 2021). The ENS, also referred as the “gut brain,” interacts with the immune and endocrine systems of the gut (Furness, 2012; Heiss and Olofsson, 2019; Aktar et al., 2020). Gut microbiota play a crucial role in the development and maintenance of the ENS (Aktar et al., 2020). For example, enteroglial cells (EGCs), which are analogous to microglia in the CNS, function as support and homeostatic cells for the GI tract. Recent evidence suggest that their development and homeostatic regulation are influenced by microbiota constitution (Morais et al., 2021). In adult mice, it was demonstrated that these EGCs are constantly replenished through a homeostatic dynamic and contribute to the overall health of the gut wall and ENS (Obata et al., 2020). However, this renewal is impacted by the gut microbiota composition which partially determines gut health on a cellular level (Obata et al., 2020). In patients with SCZ, many studies identified a crossroad between EGCs depletion, altered epithelial barrier and SCZ-related gastrointestinal disturbances, influencing SCZ development and progression (Bernstein et al., 2019; Verdugo-Meza et al., 2020). Given their trophic role, the alteration of EGCs is also related to the reduced brain levels of BDNF largely reported in SCZ (Szeligowski et al., 2020; Konturek et al., 2021). Alterations of EGCs-induced BDNF depletion have been linked to reduced levels of IL-1β through phosphorylated-c-Jun N-terminal kinase-dependent pathway, and increased phosphorylation of p38 mitogen activated protein kinase (Fukumoto et al., 2020). Interestingly, recent research has implicated EGC in the development and progression of SCZ as basins for misfolded proteins and/or prions which are transmitted to the brain through vagus nerve mediated transfer of endotoxemic molecules owing to disrupted epithelial barrier (Kaczmarczyk et al., 2017; Bernstein et al., 2019; Verdugo-Meza et al., 2020). Notably, misfolded proteins can be transmitted to the brain in conditions of irritable bowel disease wherein there are elevated levels of IL-1β and other phlogistic materials that are transmitted to the brain through communications with vagal nerve due to disrupted epithelial barrier. The consequence is SCZ-related gastrointestinal disturbances, thus promoting SCZ-like behavior (Bernstein et al., 2019; Verdugo-Meza et al., 2020). The primary connection of the nervous system to the GI tract occurs through the vagus nerve, which innervates the muscle and mucosa layers of the GI tract, thus linking the gut to the ENS (Morais et al., 2021). Mechanoreceptors sense and transmit to the CNS information regarding hormones, neurotransmitter, gut cytokines and other metabolite levels, as well as overall gut health and function through the vagus nerve (Aktar et al., 2020; Joly et al., 2021; Morais et al., 2021). The vagus nerve fibers innervate the muscle and mucosa layers of the gastrointestinal tract, detect sensory signals and then relay these signals to the CNS (Wang et al., 2007). The transmission of signals from the peripheral ends of the vagus nerve to the CNS occurs though activation of mechanoreceptors that can sense luminal volume or chemoreceptors triggered by chemical stimuli such as hormones, neurotransmitters, and metabolites such as short chain fatty acids produced by EECs, which may themselves be influenced by the gut microbiota (Morais et al., 2021).

Microglial sensomes also receive signals from outside the brain through the GBA, opening microglial implication in many more processes (Abdel-Haq et al., 2018). To normalize microglia, the GBA has increasingly emerged as a potent regulator of microglial function and dysfunction in the pathogenesis of neuropsychiatric diseases (Chen et al., 2021; Huang and Wu, 2021). Of note, the gut microbiome supplies trophic ingredients derived from the breakdown of complex carbohydrate products including short chain fatty acids (SCFAs) that cross the blood-brain barrier (BBB) through the portal circulation to regulate the maturation and function of microglia (Erny et al., 2015; Rooks and Garrett, 2016; Yilmaz et al., 2021). Other microbiome metabolites with pattern recognition receptor (PRR) capacity, such as microbe-associated molecular patterns (MAMPs), produced by the gut microbiota, can also permeate the BBB to modulate microglia (Braniste et al., 2014). The gut microbiota regulates, partially through its communication with gut-located EGCs and partially through PRRs and gut-derived MAMPs, the transmission of inflammatory information throughout the ENS (Furness, 2012). Changes in the concentration of certain molecules are sensed along the lumen, triggering signal transmission of inflammatory responses within the gut, in some cases resulting in acute inflammatory responses, such as colitis or gut dysbiosis (Kamdar et al., 2016; Qi et al., 2021), a pathological state linked to increased microglial phagocytosis in SCZ (Erny et al., 2015, 2021; Munawar et al., 2021). Moreover, the roles of Toll-like receptors (TLR)-3, 7, and 9 were found to regulate microglial activities via a series of MAMPs-independent mechanisms (Wang et al., 2018). Additionally, peripheral macrophages that interact with gut metabolites or MAMPs via gut flora-mediated signaling can cross the BBB and target microglia to regulate their activities (Wang et al., 2018).

The continuous GI tract inflammation can lead to systemic inflammation via chronically high levels of pro-inflammatory cytokines in circulation, resulting in damage throughout the body’s organs (Parker B. J. et al., 2020). Increases in systemic pro-inflammatory cytokines present in the brain causes damage to the BBB, further raising the inflammatory response as a result of increasing pathogens and toxins from the deteriorating BBB (Parker A. et al., 2020). While an imbalanced or abnormal gut microbiome can result in this runaway inflammation, a balance of beneficial gut microorganisms promotes the secretion of anti-inflammatory cytokines resulting in an overall decrease in inflammation both locally and systemically (Desbonnet et al., 2008; Dowlati et al., 2010; Sarkar et al., 2016; Peppas et al., 2021). Thus, these findings suggest that the gut microbiota is a central figure in the health of the gut, which plays a critical role in determining changes in metabolism, endocrine system, nervous system communication, and inflammatory responses, as observed in the pathophysiology of SCZ.

## 7. Emerging epigenetic changes modulating the gut-brain microglia axis

Recent studies have investigated the effects of environmental factors on epigenetic changes during human neurodevelopment. These outcomes directly modify the transcription and expression of genes including from the complement pathways—“turning on” some genes, while “turning off” others (Föcking et al., 2021; Ji et al., 2022). The complement pathway is a vital component of the immune defense against certain immune stimulating factors (Föcking et al., 2021), which include stress, infection, in- and out-door pollution, and nutrition (Figure 1; Afighor et al., 2019; Elizabeth et al., 2020; Oladapo et al., 2021; Osagie et al., 2021; Ben-Azu et al., 2022). There is emerging evidence that these environmental factors directly affect the GBA—demonstrating a mechanism inducing direct alterations during development and throughout life. Of note, early life stress and infection are key epigenetic factors that have been largely linked to the emergence of SCZ-like feature during adulthood (Giovanoli et al., 2016; Ben-Azu et al., 2019, 2020).

Recently, there has been increasing evidence suggesting an association between air pollution and intestinal diseases, specifically inflammatory bowel syndrome, appendicitis, and colorectal cancer (Li et al., 2019; Feng et al., 2020). Mounting evidence also suggest that air pollution and stress affect brain development, adversely through modification of early life microbiome, and might serve as a risk factor for developing psychiatric diseases such as SCZ due to mechanisms linked to modulation of epigenetic codifiers and readers including covalent histone modification, DNA methylation, and non-coding RNAs (David et al., 2017; Comer et al., 2020; Newbury et al., 2021). For instance, parental isolation causes phosphorylation of methyl CpG binding protein and a disassociation of DNA strands from protein moieties, thus leading to a post-translational modification of epigenetic modifiers linked to neurodevelopmental disorders (Chahrour et al., 2008). Hypermethylation of the genes glutamic acid decarboxylase (GAD) 67 (*GAD1*), which is responsible for the synthesis of the major inhibitory neurochemical, GABA, as well as reelin (*RELN*), an extracellular matrix protein involved in the regulation of neuronal migration and positioning, were reported in the brain and periphery of patients with SCZ after post-mortem investigation (Guidotti et al., 2000; Abdolmaleky et al., 2005; Huang and Akbarian, 2007; Magwai et al., 2021). Down-regulation of *RELN*, *GAD1*, or GAD67 are linked to impaired prefrontal cortical dendritic arborization and activity related to the working memory deficits of animals and patients with SCZ (Ben-Azu et al., 2018b; Magwai et al., 2021; Oshodi et al., 2021).

Certain air pollutants that include ozone, sulfur dioxide, and carbon monoxide were associated with increased inflammation in the gut, while their short-term exposure is linked to increased occurrences of appendicitis (Kaplan et al., 2009) and there seems to be a relationship between appendicitis and the occurrence of neuropsychiatric disorders such as SCZ (Isung et al., 2019; Parker B. J. et al., 2020). Moreover, anecdotal evidence suggest a high rate of appendiceal perforation in patients with SCZ vs. controls (Nishihira et al., 2017). Not only does air pollution seem to have a direct effect on the gut inflammatory status, but it was also shown across *in vivo* and *in vitro* studies to disrupt lipid metabolism, commonly resulting in increased pathological metabolites such as serum cortisol/corticosterone, monoacylglycerol, glycerol, lysolipids, mitochondrial β-oxidation-derived metabolites like acylcarnitines and ketone bodies in the serum as well as hexanoyl-lysine in the serum, liver and brain (Tomaru et al., 2007; Vesterdal et al., 2014; Miller et al., 2016). These derangements are linked to a short supply of short chain fatty acid, consequently leading to depletion of polyunsaturated fatty acid (PUFA) which are both needed for normal brain signaling. Another study also revealed that diesel exhaust particles increase the levels of hexanoyl-lysine in the liver of obese diabetic subjects as well as levels of aspartate aminotransferase (AST) and alanine transaminase (ALT) compared to that to vehicle. Of note, debilitating cerebral edema has been reported as one of the devastating consequences of acute liver damage following exposure to hepatoxins which occur due to BBB breakdown-derived oxidative stress and exacerbation of existing inflammatory milieu (Jayakumar et al., 2013; Kim et al., 2014). In addition, air pollution affects microglia-astrocyte interactions leading to exacerbated brain inflammation and oxidative stress (Gómez-Budia et al., 2020). Even though many studies examining the relationship between air pollutants and intestinal diseases are epidemiological and comprise uncontrolled, confounding variables, the general trend observed suggest a relationship between air pollution and gut diseases. Considering the crucial role of gut microbiota in determining health of the gut, the effect of pollutants on the gut microbiome is a worth-while concept of investigation notably in SCZ (Bernardini and Attademo, 2021). Many studies have suggested that exposure to chronic air pollution can up-regulate brain expression of microglial genes and pro-inflammatory cytokines such as tumor necrosis factor (TNF)α, IL-1β, and IL-6 in animals and humans following exposure to nano-particulate matter from traffic-related air pollution (Calderón-Garcidueñas et al., 2015; Gruzieva et al., 2017). It will be important to develop translational models to elucidate further how air pollution could adversely affect brain neurons, their microglia-astrocyte crosstalk, and the influence from the gut microbiota and microbiome.

Stress was found to be quite impactful in altering gut health and microbiome development. Stress signaling is primarily mediated through the hypothalamic-pituitary-adrenal (HPA) axis to the gut and has been demonstrated to lead to leaky gut, lower gut motility, and decreased microbial abundance (Keita and Söderholm, 2010; Yu et al., 2017). Exposure to chronic and acute stress in early life has been shown to reduce gut biodiversity, notably resulting in a decrease of human growth hormones in early development (Qi et al., 2021). Human studies revealed that prenatal stress and depression can alter the microbiome composition, in association with lower birth weight and preterm birth (Rondó et al., 2003; Zijlmans et al., 2015). These findings emphasize the need for further research into how to promote healthy gut microbiomes for both mother and infants, as potential therapeutics to prevent the development of SCZ.

Diet is an important factor toward microbiome health throughout the host lifetime (Singh et al., 2017; Hills et al., 2019; Johnson et al., 2019; Alemao et al., 2021). Diet restrictions and selectivity are commonly observed in children with SCZ leading to nutritional limitations and a marked decrease in gut microbiome composition (Alemao et al., 2021; Onaolapo and Onaolapo, 2021). During healthy adulthood, changes in diet can result in significant alterations of the gut microbiome within 24 h (Wright and Starkweather, 2015). The *Mediterranean* diet is commonly considered a healthily balanced diet which primarily consists of high intake of olive oil, fruits, vegetables, and nuts, moderate intake of red wine, poultry, and fish, and relatively low intake of red meat and dairy (Figure 1; De Filippis et al., 2016; Singh et al., 2017). Several studies have demonstrated associations between healthy microbiome composition and the *Mediterranean* diet, as well as opposite associations with other diets, such as the *Western* diet (low fiber, high fat, and animal protein) and gluten-free diet (Wu et al., 2011; Lopez-Legarrea et al., 2014; De Filippis et al., 2016; Singh et al., 2017). While examining the consequences of diet on the gut microbiome can be challenging, dietary fibers and polyphenols, the main active ingredient in tea, fruits and vegetables, have consistently demonstrated positive correlations with a balanced gut microbial environment (Figure 1; Zhang et al., 2021).

It has been hypothesized that SCFAs which are a chain length of 1–6 carbon atoms derived from fiber content of foods by microbiota, regulate activities between the gut microbiome and the brain (Dalile et al., 2019). Some examples of SCFAs include acetate, butyrate, propionate, formate, valerate, and caproate. Notably, acetate, butyrate and propionate are produced in very high amounts in the ratio of 60:20:20 as the most copious anions in the proximal bowel, whereas formate, valerate, and caproate are formed in lower quantities (Macfarlane and Macfarlane, 2003). The levels of SCFAs produced are based on a variety of factors such as type of diet, microbiota system and colon transition time (Macfarlane and Macfarlane, 2003; Dalile et al., 2019). Following absorption from colonocytes into the systemic circulation, SCFAs play important role in cellular ATP generation from mitochondrial citric acid cycle (Schönfeld and Wojtczak, 2016). SCFA anion reaches the brain via the expression of monocarboxylate transporter-1 by BBB endothelial cells (Vijay and Morris, 2014; Dalile et al., 2019). In addition, SCFAs modulate microglial homeostasis via a free fatty acid receptor (FFAR)-dependent signaling pathway in mice (Erny et al., 2015). Research into the associations between diet and the gut microbiome composition in determining health will be key to provide further insight into the therapeutic potential of modifying the GBA via diet, notably in SCZ.

The gut microbiome is a critical factor toward preventing infection of the GI tract (Ducarmon et al., 2019). Producing bile acids, bacteriocins and bacteriophages, which contribute to creating a competitive nutrient environment, and fortifying the GI tract’s epithelial barrier are all part of the gut microbiome’s repertoire to maintain gut homeostasis (Vollaard and Clasener, 1994; Ducarmon et al., 2019). However, many exogenous agents can affect the gut microbiome’s ability to counteract pathogens, such as antipsychotics, proton pump inhibitors, antibiotics, antidepressants and diabetic medications (Ducarmon et al., 2019; Flux and Lowry, 2020). Infections can cause a dysbiotic state of the gut microbiome characterized by a leaky gut and high inflammatory status (Clapp et al., 2017), which can increase the host vulnerability to developing neuropsychiatric diseases such as SCZ. The effects of dysbiosis on the gut and brain health particularly as it promotes accelerated brain aging and neuropsychiatric states like SCZ via mechanisms related to gut-brain microglia axis will be further investigated in the next section.

## 8. Dysbiosis-induced microglial dysfunction in SCZ and its accelerated brain aging

The role of the gut microbiome in the development and regulation of the CNS, especially via EEC-induced synthesis of neurotransmitters, but also microglial maturation, is well documented, with outcomes on the control of behavior and cognition (Erny et al., 2015, 2021; Abdel-Haq et al., 2018). Consequently, dysbiosis-induced microglial dysfunction has increasingly become an interesting aspect of the GBA (Chen et al., 2021; Huang and Wu, 2021). Consecutive administration of broad-spectrum antibiotics (cefoxitin, gentamicin, and metronidazole) to male and female mice for a month induced temporal depletion of host microbiota which was associated with markedly enlarged caeca and deficits in microglial maturation, as well as neuroimmune response (Erny et al., 2015). In line with this finding, GF conditioning was associated with low populations of bone marrow-derived splenic macrophages and monocytes (Khosravi et al., 2014). This outcome was suggested to result from reduced myeloid survival factor colony stimulated factor 1 (CSF1), thereby translating into reduced microglial function (Khosravi et al., 2014; Erny et al., 2015). Notably, microglia are seeded elements from the embryonic hematopoietic yolk sac, which enter the brain starting at embryonic day 9.5 in mice, thus reinforcing the relevance of early microbial colonization to effectively respond to pathogens later in life (Ginhoux and Prinz, 2015). In adulthood, microbiota ablation was shown to trigger hyperactive and irregular HPA activity in GF mice exposed to stress (Sudo, 2016). This aberrant response was linked to exacerbated cortisol release, translocation of gut-derived metabolic-end products and bacterial antigens across the BBB, which are associated with the pathogenesis of SCZ, notably in conjunction with microglial dysfunction (Sudo, 2016; Picard et al., 2021; Rim et al., 2022).

Gut dysbiosis-induced microglial dysfunction was also shown to be sex-dependent. For example, Thion et al. (2018) demonstrated that microglia display age-dependent sex-specific vulnerability to microbiota ablation, with male showing an early uterine manifestation and female exhibiting profound changes during adulthood. Regardless of the life stage, microglia from GF showed enhanced transcriptomic genetic signatures indicative of a premature immune state with sex-specific outcomes. The conceivable influence of the gut dysbiosis as a precursor for SCZ and accelerated aging stems from the hypothesis that lifelong cohabitation of the gut microbiota as an immune regulator can initiate dysfunctional microglia-neuron interactions following maternal immune activation (Thaiss et al., 2016; Abdel-Haq et al., 2018; Reyes et al., 2020). Importantly, identifying genera associated with increased microglial dysfunction in SCZ and accelerated aging is important for designing relevant probiotics that could help maintain a young gut microbiota. This strategy aims to slow down aging and associated neurological diseases, especially in vulnerable individuals with SCZ or advanced age groups (Xu et al., 2019).

### 8.1. Gut dysbiosis in SCZ

An increasing body of epidemiological reports has provided significant evidence for a connection between prenatal infections and increased risk for later development of neuropsychiatric disorders (Awogbindin et al., 2021). Neuroimmune activity during the first phase of life (age 1–3 years) is important for cognitive and social flexibility later in life, especially in adolescence and adulthood (Allswede et al., 2016; Kelly et al., 2021). At birth and in newborns, vaginal microbes as well as those from maternal diets and immunological complements from breast milk colonize different organs including the brain (Al Nabhani et al., 2019). Perturbations of maternal gut microbiome during early phases of life from embryonic development until weaning can impact the immune system, thereby causing a pathological “priming” or an increased immune responsivity to future challenges of microglia which are still developing (Al Nabhani et al., 2019; Rosin et al., 2021). A nationwide study of hospitalized children in Denmark (*N* = 1,015,447) between 1985 and 2002 showed a close relationship between treatment with anti-infective agents and a higher risk of developing SCZ with a hazard rate ratio of 2.05 (95%-Cl = 1.77-2-38). Evidence suggests that this enhanced vulnerability to SCZ was mediated by a dysregulated adjustment of the gut microbiome after treatment of infections with wide-spectrum antibiotics (Yolken et al., 2016; Köhler et al., 2017; Köhler-Forsberg et al., 2019). Experimental work on gut dysbiosis indicates that altered intestinal barrier coupled with dysregulated microbial populations may allow for leaking of antigenic gastrointestinal molecules causing activation of the complement system of immune cells including microglia (Lambert, 2009; Mossad and Erny, 2020). Experimentally induced immune alterations during prenatal life with antibiotics were also shown to alter the microbiota system in mice (Russell et al., 2013; Gonzalez-Perez et al., 2016; Benner et al., 2021). Additionally, studies examining neuronal functioning revealed that mice exposed to maternal immune activation with viral mimicry agents display during adolescence and adulthood SCZ- and autistic-like behavior including reduced communication and social interactions, together with increased stereotypy, anxiety and sensorimotor deficits (Coiro et al., 2015; Meehan et al., 2017; Pendyala et al., 2017; Hui et al., 2018).

### 8.2. Accelerated aging and gut microbiota

Aging is a rate-limiting factor that modifies the functional activities of different body organs. There are numerous pathways that could influence the aging rate including factors such as environment and diet, genetics and pathological conditions (Finlay et al., 2019; Kim and Benayoun, 2020; Li et al., 2021; Narasimhan et al., 2021). Increasing evidence is beginning to show that the diversity of the human gut microbiome is also correlated with aging, which is based on the aging progression of the microbiota (Xu et al., 2019). Of note, different multivariate reports hypothesize that the human aging process is determined by the continuous aging curve of gut microbiota community, dysbiosis, and depends on incidence or rate of infection, antibiotic usage, type of genera, declined metabolic activity and availability of gut metabolites including SCFAs (Lovat, 1996; Vatanen et al., 2018; Xu et al., 2019; Hendriks et al., 2021), which in turn influence brain aging (Nguyen et al., 2021). Using high throughput whole genome sequencing and metagenomics, microbial species such as *Bacteroides*, *Clostridiaceae*, and *Eubacterium* were reported to be increased during aging (Odamaki et al., 2016; Loughman et al., 2020). These species can influence neurotransmitter synthesis such as glutamate and GABA, and associated behavioral outcomes which have been largely implicated in brain aging (Dinan and Cryan, 2017; Zheng et al., 2019; Chen et al., 2021). Notably, these findings support the possibility that leaky gut during dysbiosis may permit a translocation of gut-derived metabolic-end products, enteric microbes, as well as food and bacterial antigens into systemic circulation and across the BBB (Abdel-Haq et al., 2018; Dabke et al., 2019). This potentially contributes to sustaining an inflammatory gut environment, leading to the brain physiological and structural anomalies observed in aging (Hsiao et al., 2013; Nguyen et al., 2021). Thus, these data indicate that using proteomics, metabolomics, transcriptomic, DNA methylation and telomere length analyses, the microbiota ecosystem can be used to uncover the biochemical landscape underlying the inter-organ transfer of molecules and gut-brain connections that likely promote accelerated aging (Lai et al., 2021; Nguyen et al., 2021).

Mechanistically, some correlations were identified between an increased intestinal bacterial synthesis of 3-deoxy-D-manno-octulosonic acid-lipid (Kdo2-lipid), TMAO and an accelerated disease state-induced brain aging (Zeisel and Warrier, 2017; Li et al., 2018). Notably, it was discovered that Kdo2-lipid and TMAO biosynthesis are altered in neurodegenerative diseases, in association with increased inflammatory cytokines and risk of coronary heart and IBD (Verdugo-Meza et al., 2020; Nguyen et al., 2021). Kdo2-lipid is an immune stimulant released by lipopolysaccharide (LPS) in most gut microbial metabolism that causes host immune stimulation by activating TLR-4. TMAO is generated from trimethylamine derived from foods like fish or indirectly from the bacterial breakdown of dietary phosphatidylcholine, betaine, L-carnitine in the gut as well as enteric tract cell fragments (Koukouritaki et al., 2002). Colonization of gut microbiome of gnotobiotic mice with trimethylamine-forming microbes within the cecum and colon significantly increased TMAO concentrations via flavin monooxygenases-mediated metabolism and dramatically reduced dietary choline levels, which was worsened upon increasing population of trimethylamine-forming bacteria (Chao and Zeisel, 1990; Romano et al., 2015). TMAO is an immunologically active gut-bacterial derived metabolite (Chen et al., 2019), with innate capacity to up-regulate NLRP3, caspase-1, IL-1β, IL-6, and 1L-18 activities that could lead to chronic metabolic and neuropsychiatric diseases characterized of vascular senescence and accelerated aging (Zeisel and Warrier, 2017; Ke et al., 2018; Brunt et al., 2020). Correlatively, exogenous TMAO systemic administration for 16 weeks accelerated brain aging in 24-week-old senescence accelerated prone strain 8 mice, typified by a significant number of senescent cells mitochondrial death, and oxidative stress in the hippocampus, accompanied by memory impairment (Li et al., 2018). These findings suggest possible mechanisms by which an altered gut microbiota could negatively induce the accelerated brain aging increasingly observed in neuropsychiatric diseases like SCZ. These findings also provide insight into the functional connection between the gut and brain, also proposing that TMAO could serve as a useful marker for the diagnosis accelerated aging in SCZ. Together, these findings provide insights into the gut microbiota involvement in premature brain aging and potential mechanisms to slow down senescence by modulating gut microbiome-derived metabolites acting on microglial functions.

## 9. Effects of antipsychotics on the gut microbiome: therapeutic and adverse effects

Neuroleptic drugs including typical and atypical antipsychotics are clinically prescribed for the management of SCZ and other related psychotic diseases such as conduct disorder, oppositional defiant disorder, ASD and borderline personality disorder (Cheng-Shannon et al., 2004; De Hert et al., 2011; Olfson et al., 2012). Different reports have been provided for antipsychotic-related therapeutic and adverse effects of typical and atypical antipsychotic drugs during usage in psychotic conditions. Of pertinence, some therapeutic benefits have been linked to modulation of neurochemical transmission, as well as inhibition of oxidative stress and inflammation. However, their adverse outcomes including extrapyramidal symptoms (like locomotor impairment, tremor, stiff muscle, and tardive dyskinesia) and metabolic effects such as weight gain and obesity are attributable to alterations in neurotransmitter homeostasis (Saddichha et al., 2008). Of increasing interest is the role of the gut microbiome in the therapeutic and adverse effects of antipsychotic drugs and the reciprocal influence of the gut microbiome on the pharmacokinetic profiles of antipsychotic drugs (Kraeuter et al., 2020; Singh et al., 2022).

Different studies revealed that many antipsychotic drugs change the composition of gut microbiota, either by population or depopulation, by modifying mucosal integrity and membrane permeability (Tyski, 2003; Dinan and Cryan, 2018; Lima et al., 2019; Vich Vila et al., 2020). For example, some phenothiazines including chlorpromazine, fluphenazine, and thioridazine, second generation antipsychotic drugs (risperidone, clozapine, aripiprazole, and olanzapine) exhibit intrinsic antibiotic tendency against bacterial isolates of Gram-positive and Gram-negative organisms derived from the mammalian gut (Kristiansen and Mortensen, 1987; Morgan et al., 2014; Maier et al., 2018). Remarkably, an immunosuppressive concentration-dependent action of typical (e.g., haloperidol) and atypical (e.g., clozapine) antipsychotics possibly linked to IL-1 receptor antagonism was reported (Song et al., 2000). More recent studies showed that most of these drugs inhibited similar gut microbiota species irrespective of their chemical characteristics, thus pointing to the fact that their clinical application could be dependent on the spectrum of gut microbiotic specie populated or depopulated (Maier et al., 2018; Singh et al., 2022).

In terms of pharmacodynamic effects, the gut microbiome is an important site for the synthesis of different neurohormone transmitters including dopamine, serotonin, noradrenaline, acetylcholine, while microbiota diversity may strongly affect these neurochemical levels (Roshchina, 2010; González-Arancibia et al., 2019; Saniotis et al., 2020; Szõke et al., 2020). Given that the primary mechanism of action for antipsychotic drugs is neurochemical modulation (Ben-Azu et al., 2018c,^2023^; González-Arancibia et al., 2019; Szõke et al., 2020), it is unsurprising that the gut microbiome could significantly impact the action of antipsychotic drugs in the brain, either by reducing or enhancing their effectiveness (Seeman, 2021a). Some investigations have shown that GF mice exhibited reduced neurochemical levels, such as decreased mRNA expression of NR2 subunit of *N*-methyl-*D*-aspartate receptor in the central amygdala, as well as low serotonin receptor (5-HT) 1A in the hippocampus and histamine levels in the limbic system (Neufeld et al., 2011; Clarke et al., 2013; Panula and Nuutinen, 2013; Chen and Liu, 2021). These findings reinforce the inter-organ connectivity between the gut and brain, and the potential influence of gut microbiota diversity on the pharmacodynamic profile of antipsychotic drugs. On this ground, Seeman (2021a) recommends avoiding antibiotic treatment during antipsychotic therapy, while nutritional enhancement with probiotics is recommended to improve general health and wellbeing during management of psychotic conditions.

Some preclinical and clinical experiments have also investigated the role of the gut microbiome in determining the adverse profile of antipsychotic medication (Davey et al., 2013; Morgan et al., 2014; Bahr et al., 2015). Olanzapine interacts with the gut microbiome to induce significant weight gain and adiposity in control mice, while GF mice treated with olanzapine exhibited little or no weight gain after 7 weeks. Proteomic analysis of fecal pellets of rats revealed that olanzapine causes a shift toward obesogenic bacterial phyla including *Actinobacteria*, *Alphaproteobacteria*, *Clostridia*, and *Firmicutes* (Davey et al., 2013; Morgan et al., 2014). Oral co-administration of antibiotic cocktail containing neomycin (250 mg/kg/day), metronidazole (50 mg/kg/day), and polymyxin B (9 mg/kg/day) with olanzapine dramatically prevented olanzapine-induced weight gain, uterine fat decomposition and macrophage infiltration of adipose tissue (Davey et al., 2013). Also, 16S ribosomal RNA sequencing of fecal bacteria population in children treated with risperidone showed compositional shift toward obesogenic bacteria profile particularly with increased Bacteroidetes and Firmicutes levels compared to antipsychotic naïve psychiatric group. This outcome suggests a possible link between high body mass index (BMI), weight gain and deregulated synthesis of SCFAs, as well as tryptophan metabolism in the gut during antipsychotic therapy and could be due to blockade of neurohormone receptors such as muscarinic, H1 and 5-HT2C (Jumpertz et al., 2011; Bahr et al., 2015).

Nevertheless, variable sex-dependent effects of antipsychotic drugs-induced metabolic syndrome and gut microbiota alterations have been postulated, with female rodents having higher rates of obesity and cognitive symptoms (Rubin et al., 2008; Davey et al., 2012, 2013; Morgan et al., 2014; Bahr et al., 2015; Seeman, 2021b). Although the gut microbiome and its composition differ among humans and gender due to hormonal variations and route of drug administration (Zhang et al., 2013; Kim et al., 2020; Yuan et al., 2020), microbiome-induced drug metabolism could negatively impact host’s pharmacokinetic profiles including metabolic enzymes involved in phases 1 and 2 such as cytochrome P450s (a hemeprotein involved in the metabolism of drug and xenobiotics) to determine bioavailability, efficacy and toxicity (Enright et al., 2016; Cussotto et al., 2021). A typical example is the activation of the pro-drug or chemical scission of isoxazole in the benzisoxazole ring system of risperidone to active metabolites (such as 9-hydroxy-risperidone and paliperidone) in the presence of gut microflora under aerobic and anaerobic states (Meuldermans et al., 1994; Wilson and Nicholson, 2017; Xie et al., 2020). Altogether, these findings indicate the possible influence of microbiota system on antipsychotic-induced adverse effects (weight gain, higher BMI). Thus, widening the scope to include gut microbiota profiling during antipsychotic therapy may eventually result in improved therapeutic strategies and outcomes. However, this axis remains open for elucidation of a clear connection between dysbiosis and the adverse effects of antipsychotic drugs for efficient clinical outcomes.

## 10. Do fecal microbiota transplants populate microglial sensomes and affect brain reserves?

The clinical application of the effect of interpersonal variations of microbiome on pharmacokinetics, pharmacodynamics and adverse effects of drugs known as “pharmacomicrobiomics” is increasingly emerging because of the striking evidence demonstrating that fecal transplantation of healthy microbiota decoction into dysbiotic gut of ill individuals can be beneficial. While fecal microbiota transplantation (FMT) (Figure 1) entails the seeding of healthy bacteria contained in fecal products from healthy donors to diseased individuals (Zheng et al., 2019; Chinna Meyyappan et al., 2020; Zhu et al., 2020b; Erny et al., 2021), pharmacomicrobiomics is the study of the interactions between drugs and microbiome (Sharma et al., 2019). Gut microbiota community can regulate CNS activity, which is partly dependent on genetic and epigenetic factors (Goodrich et al., 2014; Levine et al., 2018; Montgomery et al., 2020). One possible connection between the gut microbiome and brain’s immune status is the role of microglia in regulating neuro-immune responses, and brain metabolic activity, as well as the reciprocal reprogramming of microglia by the gut microbiome (Clarke et al., 2013; Abdel-Haq et al., 2018; Bernier et al., 2020; Carrier et al., 2020; Cornell et al., 2021). Transplantation of microbiota fecal materials from SCZ patients to GF mice has been reported to cause SCZ-like behavior typified by hyperlocomotion, sensorimotor gating deficit, anhedonia-like symptoms and neurochemical imbalance characterized by decreased glutamate in the hippocampus, increased cellular basal dopamine in the prefrontal cortex and serotonin levels in the hippocampus (Zheng et al., 2019; Zhu et al., 2020a). Although SCZ-like behaviors were previously linked to brain inflammation and microglial dysfunction (Morgan et al., 2010; Abdel-Haq et al., 2018), whether the microbiome-microglia brain axis directly mediates the effects of the FMT on modulating SCZ-like behavior remains to be investigated in experimental animal-human models.

Of note, translocator-positron emission tomography (PET) imaging scans of individuals at ultra-high risk of SCZ demonstrated a higher binding of radiotracer markers (such as [^11^C]PBR28 and [^11^C]^®^-PK11195 radioligands) for 18 kDa translocator protein (TSPO), a relatively non-specific marker for immune reactivity, in gray matter regions, suggesting the implication of microglia and (neuro) inflammation (Bloomfield et al., 2016; Conen et al., 2021). A study showed that GF mice displayed underdeveloped and immature microglia in the cerebral cortex, corpus callosum, hippocampus, olfactory bulb, and cerebellum with a wide array of gene expression changes pertaining to cytokines and chemokines compared to colonized SPF microglia. Using quantitative real-time PCR analysis, these changes included *S100a4*, *S100a6*, *S100a8*, and *S100a10* genes following LPS or lymphocytic choriomeningitis virus (LCMV) inflammatory induction (Erny et al., 2015). Furthermore, Erny et al. (2015) demonstrated that under GF conditions, microglial response to pathological insults such as viral exposure is less severely characterized by up-regulation of CSF1-receptor, F4/80 and CD31 surface proteins. The study also found substantial high levels of several other genes involved in the promotion of cell proliferation [e.g., *Iqgap1*, *DNA-damage inducible transcript-4* (*Ddit4*)], cell cycle (e.g., Cdk9 and Ccnd3), and apoptotic inhibition (Bcl2) in the microglia of GF mice. The microglia of GF mice displayed altered morphology characterized of increased cell division, branching, and segments, through mechanisms linked to metabolic elevated expression of *Csflr*, *Ddit4* and *Transforming growth factor beta* (*Tgf-*β) 1 genes (Erny et al., 2015; Mossad and Erny, 2020; Wang et al., 2022). Of relevance to microbiome reconstitution, these defects were partially restored by recolonization with a complex microbiota and microbiota-derived bacterial fermentation. Also, defective microglia were reversed by SCFA supplementation, promoting restoration of microglial process length, number of branching and segments (Erny et al., 2015). These findings further suggest that continuous contribution of the gut microbiome is critical for microglia-regulated functions, including neuroimmune response and behavior in steady-state conditions.

Gut microbes are essential for the release of SCFAs, which are bacterial fermentation products required to maintain intestinal immune cell homeostasis through peripheral regulatory T cells (Tregs)-transcription factor forkhead box P3 signaling (Burzyn et al., 2013; Smith et al., 2013), G-protein coupled receptor (GPCRs) or histone deacetylases (HDACs) (Samuel et al., 2008; Soliman and Rosenberger, 2011). Recent findings illustrated that cerebral Tregs-Foxp3 in rat cerebrum constitute over 15% of cerebral CD4(+) T cell compartment and higher Treg cell-associated signature genes than those present in peripheral counterpart (Xie et al., 2015). Cerebral Tregs-Foxp3 inhibits LPS-induced microglial reactivity and brain inflammation via IL-10, IL-35, CTLA4, and CD39 response pathways, suggesting immuno-surveillance and immunomodulatory roles of the gut-brain microglia Tregs-Foxp3 pathway in maintaining cerebral homeostasis (Xie et al., 2015). Furthermore, gut-brain microglia metabolic fitness is driven by essential bacteria-derived SCFAs specifically acetate through an up-regulation of brain acetyl-coenzyme (aCoA) (Mezö et al., 2020; Mossad and Erny, 2020; Erny et al., 2021). Although microbiota-derived MAMPs and FFAR2 for SCFAs binding have not been successfully shown to participate in the maintenance of microglia under homeostatic conditions (Erny et al., 2015), these recent findings emphasize that microglial maturation, differentiation and function are strongly controlled by host gut microbiome and complex molecular signatures, ensuring that their roles serve as chaperon for quick diagnosis of dysfunctional CNS activity relevant for SCZ pathogenesis (Erny et al., 2015; Abdel-Haq et al., 2018; Kelly et al., 2021; Rosin et al., 2021). These findings support the notions that microglial sensomes can be modulated and brain reserves can be increased by acting on the gut microbiome, thus supporting the gut-brain microglia axis hypothesis. In the future, other links showing specific gut microbiome complement-mediated microglia phagocytosis and altered synaptogenesis and morphogenesis are hereby required to identify novel therapeutic targets.

## 11. Experimental and clinical evidence for the influence of psychobiotics in SCZ

Psychobiotics, as an intervention that seeks to improve mental well-being through manipulation of the GB axis, may be in the form of prebiotics or probiotics. While probiotics are beneficial live bacteria, prebiotics include substances that encourage the growth and survival of probiotics. Some variations in microbiota diversity have been reported in many cases of first episode psychosis and chronic SCZ compared to healthy controls. Commonly reported microbiota strains particularly in first episode psychotic patients include: *Brucellaceae*, *Halothiobacillus*, *Micrococcineae*, *Lachnospiraceae*, and *Lactobacillaceae* which are particularly elevated in individuals suffering from social deficits due to a weak global functionality (Schwarz et al., 2018; Nguyen et al., 2021). By contrast, *Veillonellaceae* is decreased in these individuals compared to controls (Schwarz et al., 2018). Additionally, *Tropheryma*, *Halothiobacillus*, *Saccharophagus*, *Deferribacter*, *Halorubrum*, and *Lactobacillus* were elevated whereas *Nitrosospira*, *Anabaena*, and *Gallionella* decreased significantly (Schwarz et al., 2018). These findings are consistent with other reports from other first episode SCZ spectrum (He et al., 2018; Shen et al., 2018; Yuan et al., 2018). In the genus levels, patients with chronic SCZ showed increased *Anaerococcus* (*H* = 8.32; *p* = 0.007) with reduced levels of *Clostridium* (*H* = −15.9; *p* = 0.002), *Haemophilus* (*H* = −11.3; *p* = 0.004), and *Sutterella* (*H* = −12.0; *p* = 0.004) compared to controls (Nguyen et al., 2019). At the taxonomic levels, 35 taxa were differentially expressed. Among these, 33 sub-operational taxonomic units (sOTU), an operational classification model used to classify a group of closely related organisms, were from *Clostridiales* order, 1 sOTU from *Gammaproteobacteria* consisting of *Haemophilus parainfluenzae*, and 1 sOTU belonging to class *Erysipelotrichia* with unknown genus (Nguyen et al., 2019). In terms of association of microbial taxa and disease severity in relation to the psychopathology of SCZ, elevated levels of genus *Bacteroides* correlated with more depressive-like behavior, while low family levels of *Ruminococcaceae* were linked to heightened negative symptoms. In general, it is reported that the levels of phylum *Verrucomicrobia* are positively interrelated with self-reported mental wellness (Nguyen et al., 2019). Therefore, these data underscore the need to conduct more mechanistic clinical investigations for the possible application of psychobiotic in the treatment of SCZ (Nguyen et al., 2019; Zhu et al., 2020a).

A randomized double-blind placebo-control trial (*N* = 65) of probiotics consisting of a mixture of *Lactobacillus rhamnosus* strain GG and *Bifidobacterium animalis* subp. lactis strain Bb12 was conducted on DSM-IV confirmed-psychotic patients (Dickerson et al., 2014). Although no significant changes were observed in the positive and negative symptoms using their syndrome scale (PANSS) relative to control group, male patients were less vulnerable to bowel immobility during the trial period. However, another longitudinal pilot study examining the links between probiotic treatment, bowel distress and SCZ symptoms specifically reported on the antibody expressions of *Candida albicans* and *Saccharomyces Cerevisiae* which are highly abundant in SCZ (Severance et al., 2017). Severance et al. (2017) reported improvement in positive symptoms after PANSS test (*N* = 56) in male patients who were seronegative for *C. albicans*. Additionally, this study found significant decrease in the antibody levels of *C. albicans* only in males after 14 weeks of probiotic treatment. These findings suggest the possible connection between the severity of positive psychotic symptoms and seropositivity of *C. albicans* as confirmed in over 380 male patients with SCZ (Severance et al., 2017; Severance and Yolken, 2019). Consequently, this investigation reinforces the beneficial role of psychobiotics in SCZ and that repopulation and depopulation of some gut microbiota phyla might play a vital role in determining the therapeutic response to antipsychotic drugs (Seeman, 2021b). Elsewhere, multiplex immunoassay of 47 immune-related proteins in the serum of patients with chronic SCZ (*N* = 31) reported the immunomodulatory effects of probiotics (*Lactobacillus rhamnosus* and *Bifidobacterium animalis* strains) (Tomasik et al., 2015). Through *in silico* analysis, it was further demonstrated that a probiotic add-on therapy modulates the IL-17 group of cytokines in immune and gut epithelial cells, leading to increased expressions of BDNF, monocyte chemotactic protein-1 (MCP-1) and decreased von Willebrand factor, with unchanged expressions of other inflammatory proteins including T-cells relative to placebo control (Tomasik et al., 2015). Again, these findings showed the immunomodulatory potential of probiotics in ameliorating SCZ symptoms via inhibition of a leaky gut and enhancement of neurotrophic activity. Of note, the interplay between SCZ and elevated pro-inflammatory cytokines have been well characterized over the years (de Pablos et al., 2014; Köhler et al., 2017; Conen et al., 2021). Studies have shown that the gut microbiome can synthesize bioactive “immunomodulins” in the form of regulatory cytokines including IL-10 and TNFα that inhibit brain inflammation (Kemgang et al., 2014; Pendyala et al., 2017). Furthermore, while BDNF is implicated in SCZ pathology (Angelucci et al., 2005; Ben-Azu et al., 2018a), the microbial synthesis and regulation of neurotrophic factors such as BDNF which were shown to modulate synaptogenesis, neurogenesis, neuronal survival and neurochemical activities is expected to influence brain functions and clinical outcomes (Tomasik et al., 2015).

A proof of concept study examining the effect of probiotics on anxiety and depressive-like behavior associated with SCZ indicated that administration of *Bifidobacterium breve* A-1 for 4 weeks significantly attenuated the Hospital Anxiety and Depression Scale (HADS) total score by 25% (Okubo et al., 2019). This finding was associated with reduced PANSS anxiety/depression episodes and increased levels of *Parabacteroides* in the gut microbiome through mechanisms related to elevated expressions of TNF-dependent activated release of other cytokines such as IL-22 (Okubo et al., 2019). These findings raise awareness on the ability of probiotics to attenuate anhedonic-like symptoms of SCZ via an inhibition of cytokine release, leaky gut, and prevention of gut-derived bacterial antigens transfer to the brain. A preclinical investigation also revealed that treatment with probiotics containing *Akkermansia muciniphila* reversed high-fat diet (HFD)-induced cognitive dysfunction in rats (Higarza et al., 2021) as well as olanzapine-induced weight gain, metabolic and immune alterations in mice by inhibiting hepatic gluconeogenesis and reducing serum levels of cytokines (TNFα, IL-6) (Huang et al., 2021). Besides, another proof of concept exploratory study (NCT02637115) confirmed the beneficial effect of this *Akkermansia muciniphila* in overweight and obese human volunteers following daily oral administration for 3 months (Depommier et al., 2019). Thus, for better clinical outcome, these findings suggest that probiotic supplementation from dietary intervention could be used to abate metabolic adverse effects and epithelia barrier dysfunction associated with some antipsychotic medications.

Remarkably, a mechanistic study showed that post-treatment with prebiotic (*Xylooligosaccharides*), probiotic (*Lactobacillus paracasei* HII01) or symbiotic (*Xylooligosaccharides* and *Lactobacillus paracasei* HII01) in male obese-insulin resistant rats, induced by HFD for 12 weeks, significantly reversed peripheral insulin sensitivity, gut, and systemic inflammation as well as oxidative stress and apoptosis in the hippocampus. Although the superiority of prebiotic vs. probiotic supplementations on the metabolic benefits remains undecided in the field, interestingly this study reports that inhibition of gut and systemic inflammation was associated with decreased microglial reactivity, mitochondrial dysfunction, increased dendritic spine density, improved hippocampal plasticity, long-term potentiation and cognitive functions (Chunchai et al., 2018). While the inability of probiotic (*Lactobacillus rhamnosus*, JB-1) to prevent cold pressor-induced cognitive impairment in males has been reported (Kelly et al., 2017), another investigation proved that probiotics (*Lactobacilli*, *Lactococci*, and *Bifidobacteria*) in healthy females volunteers improved cognitive functions by raising the relative abundance of bacterial taxa known to protect the integrity of the intestine and BBB (Bloemendaal et al., 2021). These discrepant findings suggest that certain variables such as sex, health status, disease chronicity, microbial composition, and duration of treatment as well as genetic footprints are important factors to consider when designing studies to modulate the GB axis. Of note, the research shortcomings of using different microbial strains, genetic factors and the resilience to short-term course are discussed in-depth in the following articles (Castro-Nallar et al., 2015; Montgomery et al., 2020; Yuan et al., 2020; Bloemendaal et al., 2021; Teeuw et al., 2021).

## 12. Do psychobiotics contribute to antipsychotic-induced therapeutic benefits or adverse effects through the gut-brain microglial axis?

The aim of this review was to explore and summarize how the gut-resident bacteria can modulate molecular mechanisms of accelerated aging in SCZ through microglial signaling. While convincing evidence on the gut-microglia brain axis continue to emerge, substantial data show that dysbiotic disorders can negatively impact the vagal communication with the brain and activation of the microglial sensomes. The gut-brain microglia axis hypothesis of SCZ was stimulated by the discovery that GF mice displayed global microglial defects with abnormal phenotypes in steady and disease states, which was reversed by the introduction of a complex microbiota profile. Excessive microglial reactivity due to disease state can cause behavioral perturbations relevant to SCZ-like behavior. Definitive changes of gut microbiota and microbiome compositions may also allow to increase effectiveness and reduce the adverse effects of antipsychotic drugs by rectifying defective microglia. However, because the composition of the gut microbiome is regulated by certain dynamics such as dietary habits, aging, stress, pollution, infections, as well as other factors like genetics, sex, disease chronicity, duration of treatment, and age, the microglial sensomes and its signaling may vary as well, thus, making the hypothesis difficult to test due to interpersonal variations.

In the future, the assessment of microbiota and microbiome markers together with microglial functions can be used longitudinally as diagnostic judgment for SCZ and therapeutic benefits of antipsychotic drugs. Measures from microglial phenotypes and their sensomes can be collected and used to track the progress of antipsychotic drug idiosyncratic response, the effectiveness, and severity of adverse effects, as well as the rate of accelerated brain aging. Investigating gut microbiome-dependent microglia-mediated modulations of cytokines, chemokines, complements factors, neurogenesis, synaptic pruning, dendritic arborization, and brain reserves during treatment of SCZ patients with antipsychotic drugs may give more insights and understanding of the overall inter-organ-dependent clinical symptoms. These studies also hold the potential to provide novel treatment targets for SCZ and to slow down the accelerated brain aging. To this end, specific psychobiotic regimen could be used either as monotherapy or as adjunct or supplemented from dietary intervention to decelerate disease progression and associated accelerated brain aging. Development of scientific methods for absolute and idiosyncratic profiling and identification of the genera associated with altered gut-brain microglia axis in the pathogenesis of SCZ would, however, be recommended, to enable treatment with appropriate psychobiotics. This would be meaningful for designing relevant psychobiotics either as prebiotic, probiotic or symbiotic (a mixture of prebiotics and probiotics) as well as related dietary-enriching products such as ketogenic diets that could help in reversing SCZ and related diseases or maintain young gut microbiota system to protect vulnerable SCZ groups and improve quality of life.

## Author contributions

BB-A and M-ÈT conceptualized the review. BB-A, ER, JV, MKh, MKe, and MC wrote the manuscript. BB-A, MKh, and M-ÈT edited the manuscript. MC designed the figure. All authors contributed to the article and approved the submitted version.
